# Genomic Analysis and Antimicrobial Resistance of *Aliarcobacter cryaerophilus* Strains From German Water Poultry

**DOI:** 10.3389/fmicb.2020.01549

**Published:** 2020-07-10

**Authors:** Eva Müller, Helmut Hotzel, Christine Ahlers, Ingrid Hänel, Herbert Tomaso, Mostafa Y. Abdel-Glil

**Affiliations:** ^1^Institute of Bacterial Infections and Zoonoses (IBIZ), Friedrich-Loeffler-Institut, Federal Research Institute for Animal Health, Jena, Germany; ^2^Thuringian Animal Disease Fund, Poultry Health Service, Jena, Germany

**Keywords:** *Aliarcobacter*, *Arcobacter*, antibiotic susceptibility, whole-genome sequencing, antimicrobial resistance, virulence, plasmids

## Abstract

*Aliarcobacter cryaerophilus* (formerly *Arcobacter cryaerophilus*) is a globally emerging foodborne and zoonotic pathogen. However, little is known about the species’ genomic features and diversity, antibiotic resistance and virulence. In this study, 27 *A. cryaerophilus* strains from water poultry in Thuringia, Germany, were investigated using whole-genome sequencing. Four of these strains were sequenced using long- and short-read sequencing methods to obtain circularized genomes. The German strains belong to the *A. cryaerophilus* cluster I. Cluster I genomes exhibited a high degree of genetic diversity in which variable sites comprised 9.1% of the core genome. The German strains formed three subgroups that contained 2, 6, and 9 strains, respectively. The genomic analysis of cluster I revealed variable presence of mobile elements and that 65% of the strains lack CRISPR systems. The four circularized genomes carried a ∼2 Mbp chromosome and a single megaplasmid (size 98.1–154.5 Kbp). The chromosome was densely packed with coding sequences (∼92%) and showed inversions and shifts in the gene blocks between different strains. Antimicrobial resistance was assessed using a gradient strip diffusion method and showed that all 27 strains were resistant to cefotaxime and susceptible to erythromycin, gentamicin, and ampicillin. Sixteen strains were also resistant to ciprofloxacin, whereas 23 were resistant to streptomycin. The genetic prediction of antibiotic resistance identified numerous efflux pumps similar to those found in *A. butzleri*. All strains harbored two beta-lactamase genes which may explain the cefotaxime resistance. A correlation between the *gyr*A point mutation (Thr-85-Ile) and ciprofloxacin resistance was partially discovered in 15 out of 16 strains. *In silico* virulence profiling showed a wide range of virulence factors including a full chemotaxis system and most of the flagellar genes. In contrast to *A. butzleri*, no urease cluster was found. This study provides new insights into the genomic variability of *A. cryaerophilus* strains of cluster I. The different genetic makeup of these strains may contribute to the virulence of strains and the severity of the infections in humans.

## Introduction

*Aliarcobacter* (*A.*) *cryaerophilus* (formerly *Arcobacter cryaerophilus*) is a Gram-negative, curved motile rod that grows between 15°C and 42°C. Strains of *A. cryaerophilus* belong to cluster “1a” of the genus *Aliarcobacter* according to a recent taxonomic classification (Pérez-Cataluña et al., 2018b). *A. cryaerophilus* exhibits a high degree of heterogeneity and has been divided into two subgroups (1A and 1B) based on restriction fragment length polymorphisms of the rRNA genes, whole-cell proteins and fatty acid content (Kiehlbauch et al., 1991; Vandamme et al., 1992). However, this subgrouping was not supported using the amplified fragment length polymorphism method and the sequence analysis of the *hsp*60 gene (Debruyne et al., 2010). Recently, Pérez-Cataluña et al. (2018a) proposed the subdivision of *A. cryaerophilus* into four clusters (also called genomovars) based on whole-genome sequence analyses. The *in silico* calculation of average nucleotide identity and digital DNA-DNA hybridization indicated that these four clusters should not be assigned to a single species. *A. cryaerophilus* represents a species complex in which the four clusters represent four different species (Pérez-Cataluña et al., 2018a).

*A. cryaerophilus*, as well as other related species, namely *A. butzleri*, *A. skirrowii*, and *A. thereius*, have been associated with diseases in humans and animals (Ho et al., 2006; Collado and Figueras, 2011; Ferreira et al., 2016; Pérez-Cataluña et al., 2018a). In humans, the bacteria can cause self-limiting acute enteritis with watery diarrhea, fever and abdominal pain. A long-term study done by Vandenberg et al. (2004) showed that *A. cryaerophilus* was the seventh most common *Campylobacter*-like organism isolated from human feces. In rare cases, *A. cryaerophilus* can cause severe illnesses e.g., bacteremia (Hsueh et al., 1997). The International Commission of Microbiological Specifications for Food (ICMSF) has classified *Aliarcobacter* as a serious threat to human health in 2002 (ICMSF, 2002). Since then, *Aliarcobacter* spp. have been identified as emerging foodborne and zoonotic pathogens around the globe (Collado and Figueras, 2011; Ramees et al., 2017). In animals, *A. cryaerophilus* has been isolated from aborted fetuses and placentas of bovine, porcine, and ovine origin as well as from milk of cows with mastitis, but also from the digestive tract, feces, preputial washings, and vaginal swabs of healthy animals (Ho et al., 2006; Collado and Figueras, 2011; Ramees et al., 2017; Miller et al., 2018). Furthermore, *A. cryaerophilus* has been associated with abortion and other reproductive disorders in sows (Ramees et al., 2017).

*Aliarcobacter cryaerophilus* is present in food of animal origin such as poultry meat, dairy products and seafood as well as in drinking water and sewage (Ho et al., 2006; Millar and Raghavan, 2017; Pérez-Cataluña et al., 2018a; On et al., 2019). *Aliarcobacter* spp. are commensals in the intestinal microbiota of poultry, which can contaminate carcasses during the slaughtering process (Ho et al., 2008). Therefore poultry is a natural reservoir and acts as a major source of infection for humans (Atabay et al., 2008; Collado and Figueras, 2011). Consumption of contaminated water or food is considered as the main route of transmission to humans, while contact with companion animals is also a possible way of transmission (Ferreira et al., 2016; Ramees et al., 2017). In animals, the possibility of venereal transmission is also described as strains of *A. butzleri* and *A. cryaerophilus* have been found in the preputial washings of bulls (Ho et al., 2006).

Previous studies showed that *A. cryaerophilus* has fewer virulence-associated genes than *A. butzleri* (Ferreira et al., 2016; Brückner et al., 2020). However, limited information is currently available regarding virulence and antimicrobial susceptibility of this species (Ramees et al., 2017). Very little is known about the antimicrobial resistance and their mechanisms in *A. cryaerophilus*. Described resistance determinants are mostly located chromosomal, and no antimicrobial resistance genes have been identified on plasmids, yet. Until now, only a few studies have reported the presence of plasmids in the genus *Aliarcobacter* (Harrass et al., 1998; Douidah et al., 2014; On et al., 2019).

Here, we describe the genetic diversity and antibiotic susceptibility of 27 *A. cryaerophilus* strains isolated from seven water poultry farms in Thuringia, Germany. Furthermore, we complemented these data with 17 *A. cryaerophilus* genomes from the NCBI database and described genomic features as well as virulence-associated and antibiotic resistance genes for cluster I of the *A. cryaerophilus* complex.

## Materials and Methods

### Bacterial Strains, Culturing and Identification

In 2016 and 2017, 165 fecal samples were collected from clinically healthy animals from seven water poultry farms in Thuringia, Germany. In detail, 100 fecal samples were collected in 2016 from four water poultry farms from 50 geese (*Anser anser*), 20 Muscovy ducks (*Cairina moschata*), 20 Pekin ducks (*Anas platyrhynchos domesticus*), and ten mulard ducks (*Cairina moschata* × *Anas platyrhynchos domesticus*). In 2017, 65 fecal samples were collected from 35 geese, 15 Muscovy ducks, ten Pekin ducks and five mulard ducks from five water poultry farms. A veterinarian gathered the fecal samples with the permission of the animal owners.

For this study, no ethical review process was required, as it was no experiment with animals as defined by the German Animal Protection Law (Tierschutzgesetz) and the Animal Welfare Laboratory Animal Regulation (Tierschutz-Versuchstierordnung).

The *Aliarcobacter* isolates were cultivated in *Arcobacter* broth (Oxoid GmbH, Wesel, Germany). The broth was supplemented with three antibiotics (cefoperazone, amphotericin, and teicoplanin (CAT), Oxoid GmbH). After 48 h of incubation at 30°C under microaerophilic conditions (5% O_2_, 10% CO_2_, and 85% N_2_), the broth was spread with a 10 μL inoculation loop on plates (Mueller-Hinton agar/CAT/5% defibrinated bovine blood, Sifin GmbH, Berlin, Germany) and incubated for 24–48 h at 30°C under microaerophilic conditions. Suspicious colonies were identified by matrix-assisted laser desorption/ionization time-of-flight mass spectrometry (MALDI-TOF MS) using IVD Bacterial Test Standard and Biotyper 3.1 software (both Bruker Daltonik GmbH, Bremen, Germany) as described before (El-Ashker et al., 2015; Hänel et al., 2018). Species identification was also done with a multiplex PCR assay (Houf et al., 2000) and sequencing of the PCR products. The DNA was extracted using the High Pure PCR Template Preparation Kit (Roche Diagnostics GmbH, Mannheim, Germany) following the manufacturer‘s instructions.

### Antimicrobial Susceptibility Testing

Antibiotic susceptibility was determined by using the gradient strip diffusion method (*E*-Test^TM^, bioMérieux, Nürtingen, Germany) following the manufacturer’s instructions. Briefly, the *Aliarcobacter* strains were incubated on Mueller-Hinton agar plates (Sifin GmbH) for 48 h at 30°C under microaerophilic conditions. The colony material was put into five mL *Arcobacter* broth (Oxoid) and incubated at 30°C under microaerophilic conditions for 48 h. Then, the optical density of the broth was adjusted to 0.08 ± 0.02 at λ = 588 nm. Next, 750 μL of the broth was spread on each Mueller-Hinton agar plate (Sifin GmbH), and the antibiotic gradient strips were placed on the plates. The following antibiotics were used for testing: erythromycin (0,015–256 μg/mL, MA0108F, Oxoid GmbH), ciprofloxacin (0,002–32 μg/mL, MA0104F, Oxoid GmbH), streptomycin (0,064–1024 μg/mL, 526800, bioMérieux), gentamicin (0,06–1024 μg/mL, MA0117F, Oxoid GmbH), tetracycline (0,015–256 μg/mL, MA0105F, Oxoid GmbH), doxycycline (0,016–256 μg/mL, 412328, bioMérieux), ampicillin (0,016–256 μg/mL, 412253, bioMérieux) and cefotaxime (0,002–32 μg/mL, 412281, bioMérieux). The minimum inhibitory concentration was determined after 48 h of incubation at 30°C under microaerophilic conditions. The *A. cryaerophilus* type strain DSM 7289 was used as a control. Cut-off values for *Campylobacter* spp. provided by EUCAST (2019) were used for erythromycin, ciprofloxacin, doxycycline, and tetracycline. For gentamicin, ampicillin and cefotaxime we used the breakpoints for *Enterobacterales* from EUCAST (2019). For streptomycin, the cut-off values for *Campylobacter* spp. provided in the EFSA Journal were used (European Food Safety Authority et al., 2019). The bacterial strains were classified as sensitive (S) or resistant (R).

### DNA Extraction and Whole-Genome Sequencing

The DNA extraction was performed for 27 *A. cryaerophilus* isolates. Colony material of one to two *Aliarcobacter* culture plates was needed to obtain sufficient material for DNA preparation. The plates were washed with two milliliters of phosphate-buffered saline (PBS) and the liquid was collected in a 2-mL tube. The tubes were centrifuged for 15 min at 5,400 rpm, then the supernatant was discarded. The remaining content was washed at least twice with PBS until the supernatant was clear. The resulting pellet was further processed for DNA recovery using the QIAGEN Genomic-tip 20/G (Qiagen GmbH, Hilden, Germany) following the manufacturer’s instructions. The concentration of the double-stranded DNA (dsDNA) was examined with Qubit 3 Fluorometer using the Qubit^TM^ dsDNA HS Assay Kit (both Invitrogen^TM^, Thermo Fisher Scientific, Berlin, Germany). The Nextera XT DNA Library Preparation Kit (Illumina Inc., San Diego, CA, United States) was used to generate a paired-end sequencing library according to the manufacturer’s instructions. Whole-genome sequencing was done with an Illumina MiSeq platform generating reads of 300 bp in length (Illumina Inc.).

For plasmid DNA extraction, the QIAGEN Plasmid Mini Kit (Qiagen GmbH) was used according to the manufacturer’s instructions. The obtained plasmid DNA was dissolved in 10 μL Tris-hydroxymethyl-aminomethane (TRIS) buffer (10 mM, pH 8.3) and visualized using 1% agarose gel electrophoresis. As a size marker, 5 μL of the λDNA/*Hin*dIII Digest (Jena Bioscience GmbH, Jena, Germany) was used.

The *A. cryaerophilus* isolates in which a plasmid was detected and another strain from our sample collection (total = 4) were further investigated using the Oxford Nanopore Technology (ONT) MinION. For this purpose DNA was purified with the QIAGEN Genomic-tip 100/G (Qiagen GmbH). Sequencing libraries for ONT MinION was prepared using the ONT 1D Ligation Sequencing Kit (SQK-LSK109) with the Native Barcoding Expansion Kit (EXP-NBD104) as recommended by the manufacturer.

### Bioinformatics Analyses

Raw data from the Illumina MiSeq sequencer were assembled using shovill v1.04^1^ with options for trimming and filtering enabled (–trim, –minlen 500, –mincov 3). For the ONT data, the raw FAST5 files were processed using Guppy_basecaller v3.4.1 with high-accuracy models (dna_r9.4.1_450bps_hac) for base calling, followed by Guppy_barcoder v3.4.1 for demultiplexing. Long-read only assembly was performed using Flye v2.6 (Kolmogorov et al., 2019). Assembly polishing was performed with several rounds of Racon v1.4.3 (Vaser et al., 2017) and Medaka v0.10.0^2^. Pilon v1.23 (Walker et al., 2014) was used to correct the assembled data from ONT with Illumina reads using standard settings.

For genome annotation, the software Prokka v1.14.5 was used in default settings (Seemann, 2014). Prediction of Clustered Regularly Interspaced Short Palindromic Repeats (CRISPR) was done using the CRISPR Recognition Tool in the Geneious prime^®^ 2019.2.3 software (Kearse et al., 2012). A search for Insertion sequences (IS) and genomic islands (GI) was done using ISEscan v1.5.4 (Xie and Tang, 2017) and Islandviewer4 (Bertelli et al., 2017), respectively. Prophages were predicted using prophage_hunter (Song et al., 2019).

The 16S rRNA genes were extracted using barrnap v0.9^3^ and aligned using mafft v7.307 (Katoh et al., 2002). The program Mega X (Kumar et al., 2018) was used for the phylogenetic analysis of the 16S rRNA sequences. The Average Nucleotide Identity (ANI) was calculated using pyani v0.2.3 (module ANIm) (Pritchard et al., 2016). *In silico* DNA-DNA hybridization (DDH) was done using Genome-to-Genome Distance Calculator software (Meier-Kolthoff et al., 2013). Multilocus sequence typing (MLST) was done using the mlst tool v2.15.2^4^ and the PubMLST database (Jolley and Maiden, 2010) with default settings. Core genome-based phylogeny was performed using Parsnp v1.2 within Harvest suite with default parameters (Treangen et al., 2014). Genome comparison was carried out using progressiveMauve (Darling et al., 2010).

Public antimicrobial resistance (AMR) databases were searched for resistance-associated genes using ABRicate v0.8.10^5^ which uses the BLASTN algorithm to search AMR databases e.g., ResFinder, CARD, ARG-ANNOT, and NCBI [PRJNA313047] (Zankari et al., 2012; Gupta et al., 2014; Jia et al., 2017; Feldgarden et al., 2019).

For further investigations, the virulence and antimicrobial resistance determinants from the corresponding *A. butzleri* strains described by Isidro et al. (2020) were extracted with Geneious Prime^®^ 2019.2.3 (Kearse et al., 2012). All extracted genes were grouped in a custom database and searched within the genomes. For that, a pangenome was constructed for all strains using Roary v3.12.0 (options -i 90 -s) (Page et al., 2015). Then, the sequences of the pangenome were BLASTed against the custom database from Isidro et al. (2020) using BLASTP (Altschul et al., 1990) with the following thresholds: coverage 70% and *E*-value 1–20e. BLAST hits with more than 40% identity at the protein level were reported (Pearson, 2013).

Furthermore, the virulence-associated genes that were first found in a plasmid from an *A. cryaerophilus* isolate from a New Zealand mussel (On et al., 2019), were excised using Geneious Prime^®^ 2019.2.3 (Kearse et al., 2012) and put together into a custom database within ABRicate. The sequences used in this study were screened for those genes with a detection value of more than 30% coverage and 85% identity.

## Results and Discussion

### Bacterial Strains and Whole-Genome Sequencing

Out of 165 fecal samples, 14 were positive for *A. cryaerophilus*, nine in 2016 and five in 2017. These were obtained from ten geese, two Pekin ducks, one Muscovy duck, and one mulard duck (Table 1). Due to the different morphology of *A. cryaerophilus* on the culture plates, one to five single colonies were picked and processed separately. In total, 27 *A. cryaerophilus* strains were recovered. MALDI-TOF MS and PCR identified these isolates as *Aliarcobacter cryaerophilus*.

**TABLE 1:** The metadata of 44 *A. cryaerophilus* strains of cluster I used in this study.

**WGS**	**Strain**	**BioProject**	**Submitter**	**Isolation source**	**Year of isolation**	**Geographic location**	**Farm Nr.** – Sample Nr.*
SRR11794137	16CS0366-1-AR-1	PRJNA632720	Friedrich-Loeffler-Institut	feces (goose)	2016	Germany: Tanna	A – 1
SRR11794136	16CS0366-1-AR-2	PRJNA632720	Friedrich-Loeffler-Institut	feces (goose)	2016	Germany: Tanna	A – 1
SRR11794125	16CS0366-1-AR-3	PRJNA632720	Friedrich-Loeffler-Institut	feces (goose)	2016	Germany: Tanna	A – 1
SRR11794117	16CS0366-1-AR-4	PRJNA632720	Friedrich-Loeffler-Institut	feces (goose)	2016	Germany: Tanna	A – 1
SRR11794116	16CS0369-1-AR-1	PRJNA632720	Friedrich-Loeffler-Institut	feces (goose)	2016	Germany: Tanna	A – 2
SRR11794115	16CS0369-1-AR-4	PRJNA632720	Friedrich-Loeffler-Institut	feces (goose)	2016	Germany: Tanna	A – 2
SRR11794114	16CS0814-1	PRJNA632720	Friedrich-Loeffler-Institut	feces (goose)	2016	Germany: Uhlstädt-Kirchhasel	B – 3
SRR11794113	16CS0830-1	PRJNA632720	Friedrich-Loeffler-Institut	feces (goose)	2016	Germany: Tanna	A – 4
SRR11794112	16CS0847-1	PRJNA632720	Friedrich-Loeffler-Institut	feces (goose)	2016	Germany: Schorba	C – 5
SRR11794111	16CS0847-2	PRJNA632720	Friedrich-Loeffler-Institut	feces (goose)	2016	Germany: Schorba	C – 5
SRR11794135	16CS0847-4	PRJNA632720	Friedrich-Loeffler-Institut	feces (goose)	2016	Germany: Schorba	C – 5
SRR11794134	16CS0847-5	PRJNA632720	Friedrich-Loeffler-Institut	feces (goose)	2016	Germany: Schorba	C – 5
SRR11794133	16CS0847-6	PRJNA632720	Friedrich-Loeffler-Institut	feces (goose)	2016	Germany: Schorba	C – 5
SRR11794132	16CS1043-1	PRJNA632720	Friedrich-Loeffler-Institut	feces (pekin duck)	2016	Germany: Tanna	A – 6
SRR11794131	16CS1285-3	PRJNA632720	Friedrich-Loeffler-Institut	feces (mulard duck)	2016	Germany: Kyffhäuserland, Seega	D – 7
SRR11794130	16CS1285-4	PRJNA632720	Friedrich-Loeffler-Institut	feces (mulard duck)	2016	Germany: Kyffhäuserland, Seega	D – 7
SRR11794129	16CS1290-1	PRJNA632720	Friedrich-Loeffler-Institut	feces (goose)	2016	Germany: Kyffhäuserland, Seega	D – 8
SRR11794128	16CS1292-3	PRJNA632720	Friedrich-Loeffler-Institut	feces (goose)	2016	Germany: Kyffhäuserland, Seega	D – 9
SRR11794127	16CS1292-4	PRJNA632720	Friedrich-Loeffler-Institut	feces (goose)	2016	Germany: Kyffhäuserland, Seega	D – 9
SRR11794126	17CS0830-1	PRJNA632720	Friedrich-Loeffler-Institut	feces (goose)	2017	Germany: Schorba	C – 10
SRR11794124	17CS0996-A	PRJNA632720	Friedrich-Loeffler-Institut	feces (muscovy duck)	2017	Germany: Remda-Teichel	E – 11
SRR11794123	17CS0996-B	PRJNA632720	Friedrich-Loeffler-Institut	feces (muscovy duck)	2017	Germany: Remda-Teichel	E – 11
SRR11794122	17CS1055-A	PRJNA632720	Friedrich-Loeffler-Institut	feces (goose)	2017	Germany: Grabfeld, Wolfmannshausen	F – 12
SRR11794121	17CS1055-B	PRJNA632720	Friedrich-Loeffler-Institut	feces (goose)	2017	Germany: Grabfeld, Wolfmannshausen	F – 12
SRR11794120	17CS1061	PRJNA632720	Friedrich-Loeffler-Institut	feces (goose)	2017	Germany: Freienbessingen	G – 13
SRR11794119	17CS1201-1	PRJNA632720	Friedrich-Loeffler-Institut	feces (pekin duck)	2017	Germany: Tanna	A – 14
SRR11794118	17CS1201-2	PRJNA632720	Friedrich-Loeffler-Institut	feces (pekin duck)	2017	Germany: Tanna	A – 14
NZ_CP032825.1	ATCC 49615	PRJNA66819	USDA, ARS, WRRC	human blood	-	United States	–
GCF_002080085.1	AZT-1	PRJNA302819	Portland State University	wastewater	2013	United States: Tucson, Arizona	–
GCF_001572865.1	L397	PRJNA307600	Agriculture and Agri-Food Canada	wastewater	2008	Canada: Lethbridge, Alberta	–
GCF_001572855.1	L398	PRJNA307600	Agriculture and Agri-Food Canada	water	2008	Canada: Oldman River, Alberta	–
GCF_001573015.1	L399	PRJNA307600	Agriculture and Agri-Food Canada	wastewater	2008	Canada: Lethbridge, Alberta	–
GCF_001573005.1	L400	PRJNA307600	Agriculture and Agri-Food Canada	wastewater	2008	Canada: Lethbridge, Alberta	–
GCF_001572845.1	L401	PRJNA307600	Agriculture and Agri-Food Canada	feces (goose)	2009	Canada: Levit, Alberta	–
GCF_001572875.1	L406	PRJNA307600	Agriculture and Agri-Food Canada	water	2008	Canada: Indian Farm Creek, Alberta	–
GCF_006503595.1	123	PRJNA294644	Ghent University	feces (dog)	2006	Belgium	–
GCF_006503545.1	151	PRJNA294645	Ghent University	feces (human)	2005	Switzerland	–
GCF_006503605.1	382	PRJNA294646	Ghent University	feces (human)	2008	Belgium	–
GCF_006503615.1	938	PRJNA308312	Ghent University	feces (human)	2013	Belgium	–
GCF_006508135.1	LMG 10228	PRJNA294642	Ghent University	tissue of aborted porcine fetus	1987	Canada	–
GCF_002993045.1	LMG 10229	PRJNA369468	Universitat Rovira i Virgili	aborted porcine fetus	1990	Canada	–
GCF_002993065.1	LMG 9861	PRJNA369468	Universitat Rovira i Virgili	peritoneum of aborted bovine fetus	1990	Ireland	–
GCF_008086605.1	G18RTA	PRJNA431460	Lincoln University	shellfish (*Perna canaliculus*)	2016	New Zealand: Canterbury	–
GCF_008086685.1	M830A	PRJNA431460	Lincoln University	shellfish (*Perna canaliculus*)	2016	New Zealand: Canterbury	–

In the present study, whole-genome sequencing of 27 *A. cryaerophilus* strains was performed. The Illumina sequencing yielded an average number of 0.9 million reads per strain and an average depth of coverage 86.4X. High-quality genome assemblies were obtained except for three genomes. Those showed high contig numbers and low N50 values (Table 2). For the other 24 strains, an average N50 value of 213.3 Kbp and an average of 36 contigs per strain was calculated (Table 2).

**TABLE 2:** Sequencing, assembly statistics and annotation of 27 German *A. cryaerophilus* strains.

**Strain**	**Sequencing statistics**					**Assembly statistics**			**Annotation**		
	**Sequencing platform**	**Total number of reads (× 1000)**	**Total number of sequences (Mbp)**	**Average read length (bp)**	**Coverage depth (*X*)**	**Genome size**	**N. contigs**	**N50**	**Total CDS**	**rRNA**	**tRNA**
16CS0369-1-AR-4	Illumina MiSeq+	1088.5	247.5	227 (35–301)	121	Chromosome: 2.02 Mbp	–	–	2047	15	49
	ONT MinION	110.0	1,590.7	14,459.9 (33–285,487)	240 (chromosome) 87 (plasmid)	Plasmid: 154.49 Kbp	–	–	135		2
16CS0830-1	Illumina MiSeq +	913.7	200.8	219 (35–301)	98	Chromosome: 2.05 Mbp	–	–	2056	15	49
	ONT MinION	96.5	1,597.8	16,557.5 (27–247,704)	239 (chromosome) 105 (plasmid)	Plasmid: 128.99 Kbp	–	–	129	–	–
16CS1285-4	Illumina MiSeq +	273.5	56.4	206 (35–301)	27	Chromosome: 2.13 Mbp	–	–	2215	15	50
	ONT MinION	231.1	1,646.1	712.1 (56–206,180)	229 (chromosome) 147 (plasmid)	Plasmid: 98.09 Kbp	–	–	125	–	–
16CS1292-4	Illumina MiSeq+	889.9	200.3	225 (35–301)	98	Chromosome: 2.02 Mbp	–	–	2044	15	50
	ONT MinION	150.7	2,223.4	14,758.7 (37–223,549)	241 (chromosome) 123 (plasmid)	Plasmid: 137.35 Kbp	–	–	135	–	–
16CS0366-1-AR-1	Illumina MiSeq	504.9	118.7	235 (35–301)	58	2.15 Mbp	31	327,568	2,168	3	43
16CS0366-1-AR-2	Illumina MiSeq	734.6	167.5	228 (35–301)	82	2.15 Mbp	32	327,568	2,170	3	43
16CS0366-1-AR-3	Illumina MiSeq	481.2	115.9	240 (35–301)	56	2.15 Mbp	35	210,780	2,167	3	43
16CS0366-1-AR-4	Illumina MiSeq	735.1	167.0	227 (35–301)	81	2.14 Mbp	31	231,260	2,165	3	42
16CS0369-1-AR-1	Illumina MiSeq	667.9	151.4	226 (35–301)	74	2.15 Mbp	31	327,568	2,167	3	43
16CS0814-1	Illumina MiSeq	1,954.9	277.9	142 (35–301)	136	2.06 Mbp	39	125,155	2,092	3	41
17CS0830-1	Illumina MiSeq	443.6	112.6	253 (35–301)	55	2.09 Mbp	38	185,374	2,108	3	41
16CS0847-1	Illumina MiSeq	438.4	107.2	244 (35–301)	52	2.2 Mbp	114	40,445	2,228	3	42
16CS0847-2	Illumina MiSeq	679.3	156.7	230 (35–301)	76	2.11 Mbp	40	130,189	2,141	3	42
16CS0847-4	Illumina MiSeq	1,362.4	279.8	205 (35–301)	137	2.11 Mbp	43	113,538	2,139	3	42
16CS0847-5	Illumina MiSeq	429.1	60.4	140 (35–301)	29	2.08 Mbp	179	24,590	2,087	3	42
16CS0847-6	Illumina MiSeq	667.6	155.1	232 (35–301)	76	2.11 Mbp	41	119,731	2,138	3	42
16CS1043-1	Illumina MiSeq	2,241.7	300.7	134 (35–301)	147	2.09 Mbp	56	124,529	2,099	3	42
16CS1285-3	Illumina MiSeq	577.0	134.5	233 (35–301)	65	2.35 Mbp	58	117,068	2,354	3	40
16CS1290-1	Illumina MiSeq	552.5	139.4	252 (35–301)	68	2.11 Mbp	38	174,358	2,141	3	42
16CS1292-3	Illumina MiSeq	1,453.5	290.9	200 (35–301)	142	2.14 Mbp	63	134,716	2,164	3	43
17CS0996-A	Illumina MiSeq	738.8	183.8	248 (35–301)	90	2.02 Mbp	18	371,261	2,035	3	41
17CS0996-B	Illumina MiSeq	937.6	211.2	225 (35–301)	103	2.02 Mbp	16	371,261	2,033	3	41
17CS1055-A	Illumina MiSeq	1,203.4	269.2	223 (35–301)	131	2.16 Mbp	27	178,188	2,189	3	41
17CS1055-B	Illumina MiSeq	852.5	199.6	234 (35–301)	97	2.12 Mbp	17	326,991	2,153	3	41
17CS1061	Illumina MiSeq	750.8	169.3	225 (35–301)	82	1.96 Mbp	30	223,789	1,987	3	41
17CS1201-1	Illumina MiSeq	466.9	47.3	101 (35–301)	23	2 Mbp	540	5,487	1,926	3	30
17CS1201-2	Illumina MiSeq	1,191.5	261.6	219 (35–301)	128	2.19 Mbp	36	144,802	2,208	3	41

### Taxonomic Classification of *A. cryaerophilus* From Germany

**FIGURE 1:**
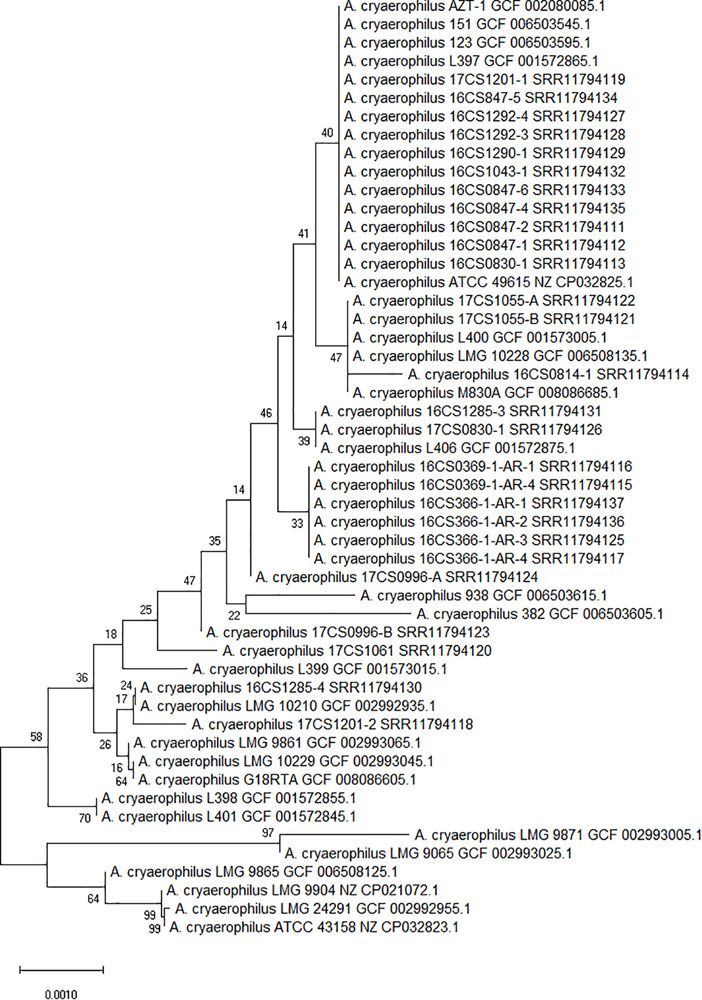
Phylogenetic tree constructed with 51 *A. cryaerophilus* strains based on the extracted 16S rRNA genes (1,520 bp). Numbers at tree branches denote the bootstrap value. Scale bar indicates the base substitution per site.

The 27 strains sequenced in this study were taxonomically classified as *A. cryaerophilus* at species level using the 16S rRNA gene (Figure 1). The 16S rRNA genes (1,520 bp) extracted from all strains were 99% identical to the 16S rRNA gene from the reference genome ATCC 43158^T^ (accession: NZ_CP032823.1), representing the type strain of *A. cryaerophilus*. Based on 16S rRNA gene analysis, the closest related species was *A. trophiarum*, with 98.6% identity. Additionally, ANI was calculated between each genome pair based on the whole-genome sequences. Results showed that the 27 German strains were highly similar (>95%) to cluster I genomes, exhibiting an average pairwise ANI of 98.1% (range 96.6–99.00%). The *in silico* DDH was additionally ascertained, showing DDH values higher than 70% when comparing the German strains to the cluster I reference genome LMG 10229^T^ (accession: GCF_002993045.1) (Supplementary Table S2). These DDH values dropped to less than 70% when the German strains were compared to the reference strains from clusters II (LMG 9065^T^; accession: GCF_002993025.1), III (LMG 24291^T^; accession: GCF_002992955.1) and IV (LMG 10210; accession: GCF_002992935.1) (Supplementary Table S2). Based on these results, we concluded that the investigated *A. cryaerophilus* strains from Germany belong to cluster I, also named *A. cryaerophilus gv. pseudocryaerophilus* based on the updated taxonomy proposal (Pérez-Cataluña et al., 2018a, b).

**FIGURE 2:**
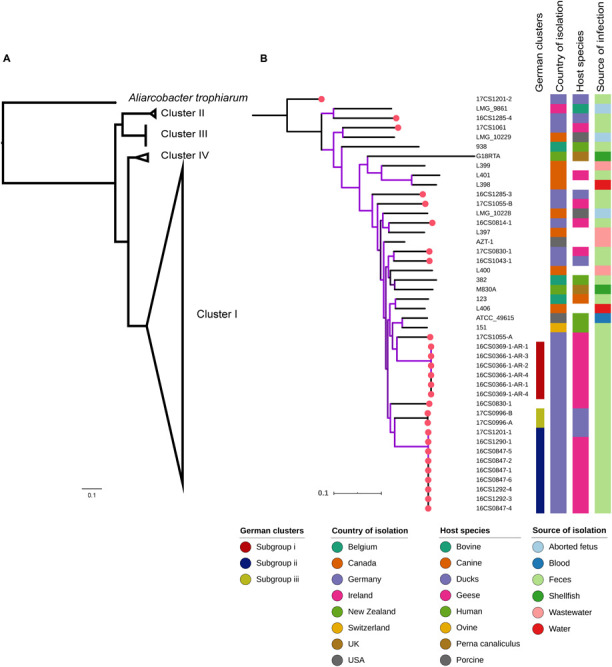
**(A)** Core-genome based phylogenetic tree depicting clusters I to IV of *A. cryaerophilus.* **(B)** Phylogenetic analysis involving 44 *A. cryaerophilus* strains of cluster I with associated metadata. The German strains formed three subgroups (i; ii; iii). Red dots indicate the German strains.

Currently, 24 *A. cryaerophilus* genomes are available at the NCBI GenBank database (Benson et al., 2013)^6^. The NCBI genomes represent strains from cluster I to IV and were collected from different hosts in different countries. Of those, 17 strains were assigned to Cluster I comprising one circularized genome (ATCC 49615; accession: NZ_CP032825.1) (Miller et al., 2018) and 16 fragmented draft genomes (average N50: 256 Kbp; average contig number: 80) (Supplementary Table S1). These strains were reported in different host species including humans (*n* = 4), pigs (*n* = 2), cattle (*n* = 1), shellfish (*n* = 2), goose (*n* = 1), dog (*n* = 1), water (*n* = 2), and wastewater (*n* = 4) (Table 1 and Figure 2). The strains span a period between 1987 and 2016 and were isolated from different countries (United States = 2, Canada = 8, Switzerland = 1, Belgium = 3, Ireland = 1, and New Zealand = 2).

### A High Genetic Diversity Between *A. cryaerophilus* From Germany Despite Limited Sources of Strain Isolation

In order to investigate phylogenetic relatedness of cluster I genomes of *A. cryaerophilus* (*n* = 44; 27 sequenced herein and 17 available at the NCBI), a core genome of 906.9 Kbp was identified. Variable sites in this cluster comprise 9.1% (82,531 SNPs) of the estimated core genome. The size of the core genomes was reduced to 579.6 Kbp when all genomes from the four clusters (*n* = 52) were taken into consideration. Of these, 11.5% (67,232 SNPs) comprise variable sites. Figure 2A visualizes the four main clusters of *A. cryaerophilus*, with most of the tree branches presenting high bootstraps values.

Based on the core genome analysis, the 27 isolates from German waterfowls (which represent seven farms in seven different places in Thuringia, see Table 1) were assigned to cluster I. In this cluster, the mean pairwise genetic distance between the German strains was 10,773 SNPs. However, three subgroups (i, ii, and iii) including 17 strains could be depicted (Figure 2B). These were mostly the strains that had been isolated from a single sample from the same farm. The subgroup i includes six strains retrieved from two fecal samples from ducks that were collected from a farm in Tanna in 2016. Two additional fecal samples were collected at the same time from the same farm, but the strains recovered were distant by more than 10,000 SNPs to subgroup i, and to each other by 10,862 SNPs. The same farm had been sampled once more in 2017, and from this additional sample, two strains were recovered. They were ∼20,000 SNPs distant from each other. In fact, one of these two strains, namely 17CS1201-1, grouped with strains from different localities in subgroup ii, with only six to seven SNP differences. The observation that strains recovered from the same sample can be highly divergent was also found in 1) two strains from Grabfeld, Wolfmannshausen isolated in 2017, in which 11,903 SNPs were detected; and 2) two strains from Kyffhaeuserland, Seega isolated in 2016, with 20,531 SNPs difference. Subgroup ii surprisingly included strains (*n* = 9) that were recovered from fecal samples collected from three farms in three different cities, five from Schorba (one sample in 2016 from a goose), three from Kyffhaeuserland, Seega (two samples in 2016 from geese) and one from Tanna (one sample in 2016 from a duck). Subgroup iii included two strains recovered from one sample from a duck farm in Remda-Teichel in 2017.

Additionally, MLST was performed based on the whole-genome sequences. All 44 genomes of cluster I were assigned to new sequence types (ST) (Supplementary Table S3). The presence of paralogs for *gly*A (i.e., multiple copies in the same genome) precluded the proper designation of STs for some of the genomes (*n* = 3). The *glyA* paralogs were identical in two genomes [17CS1055-B and L401(accession: GCF_001572845.1)] but showed variants in one genome (ATCC 49165). Isidro et al. (2020) also observed this for the *glyA* gene in strains of the species *A. butzleri*. Thus, this specific locus may not be suitable for MLST typing because it may lead to an incorrect allele calling as it has been reported for *Acinetobacter baumanii* (Gaiarsa et al., 2019). Further, we observed the absence of two MLST loci in three genomes, L397 (accession: GCF_001572865.1) lacked *glyA*, while 938 (accession: GCF_006503615.1) and AZT-1 (accession: GCF_002080085.1) both missed the *gltA* locus. It has to be noted that the quality of the assembly may influence the detection of loci. Therefore, the absence of these loci could not be confirmed. The PubMLST database (pubmlst.org, accessed on 11.02.2020) lists sequence data of 118 *A. cryaerophilus* isolates from 11 different countries, with no MLST data currently available from Germany. Those 118 strains were typed into 99 STs indicating a high genetic diversity as explained before for the core genome analysis.

These results indicate a high degree of genetic diversity among *A. cryaerophilus* strains. This was observed although the collection of the 27 strains investigated herein was restricted to one federal state (Thuringia, Germany), a particular host (water poultry) and a short study period (2 years). The strain diversity within this species is independent of the host species, as similar isolates were detected in geese and ducks (e.g., as observed in subgroup ii). This was also reflected by the global phylogeny of cluster I, in which no major clade could be identified based on the host species. Similarly, the phylogenetic analysis did not support distinct clustering based on the geographical or ecological niche of the strains. Nonetheless, it was striking that highly similar strains exist in different farms located in different places, a finding that indicates a possible epidemiological connection between these farms which might be a common source of animals. The farmer informed us that a single company in Germany supplies most of the geese-fattening farms farms in Thuringia with young animals. Muscovy ducks and mulard ducks are bred and imported from France. They are coming to Germany from a rearing farm that raises them until they are about 3 weeks old. This distributor, in turn, sells the raised animals to the fattening farms.

### Genomic Description of Cluster I *A. cryaerophilus* Strains Employing Circularized Genomes

As mentioned above, the German *A. cryaerophilus* strains belong to cluster I. To investigate the genomic features of this cluster, we focused on closing four selected genomes of *A. cryaerophilus*, as only a single genome (ATCC 49615) from this cluster had been circularized. The strains were selected based on their plasmid content as determined using a conventional plasmid detection kit (see section “Materials and Methods”). We sequenced four *A. cryaerophilus* strains additionally with the ONT sequencing method. The genome assemblies were polished with Illumina reads to improve sequence accuracy (see section “Materials and Methods”). The four genomes were composed of a single chromosome and a single megaplasmid. The size of the megaplasmids ranged from 98.1 to 154.5 Kbp with a GC content between 24.8 and 25.7% (Table 2). These plasmids carried 122–145 coding sequences, 70% thereof were hypothetical proteins.

**FIGURE 3:**
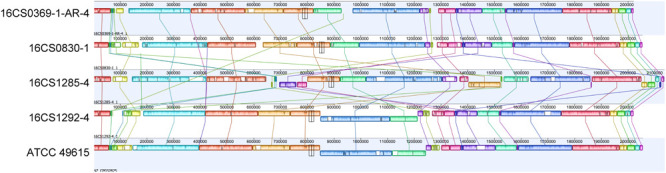
Alignment of the chromosomes from our four circularized strains together with strain ATCC 49615. The order of gene blocks was similar in 16CS0369-1-AR-4 and 16CS0830-1. In the strains 16CS01292-4 and ATCC 49615 was an inversion of a single LCB observed around the terminus of replication. Strain 16CS1285-4 had undergone several rearrangements of the chromosomal LCBs.

In the five circularized genomes (four sequenced herein and one public available), the chromosome structure was found to be consistent in terms of length, GC content, RNA genes and coding capacity. The chromosome was approximately 2 Mbp long (range: 2.02–2.14 Mbp) and had a GC content of 27.5% (range: 27.51–27.68%). Each chromosome carried five rRNA operons comprising three successive genes (16S, 23S, and 5S rRNA genes); and 50 tRNA genes (range: 49–51). A striking feature was that the chromosome was densely packed with coding sequences, with an average of 2,110 CDS (range: 2,055–2,243) that represent 91.5–93.4% of the chromosome size. This was nearly similar to *Campylobacter jejuni*, in which 94.3% of the genome code for proteins and was reported to be the densest bacterial genome reported to date (Parkhill et al., 2000). Additionally, the alignment of the chromosome from our four strains together with strain ATCC 49615 identified a considerable degree of synteny between the strains (Figure 3). The order of gene blocks (locally collinear blocks; LCB) was similar in two strains (16CS0369-1-AR-4 and 16CS0830-1) while an inversion of a single LCB was observed around the terminus of replication in the strains 16CS01292-4 and ATCC 49615. The strain 16CS1285-4 was found to have undergone several rearrangement events of the chromosomal LCBs.

Mobile element proteins (IS, GI, and prophages) were found to constitute a small part of the genome except for one strain (16CS1285-4; Supplementary Table S4). Interestingly, this strain showed several rearrangements in the homolog gene blocks. In the strains ATCC 49615, 16CS0369-1-AR-4, 16CS0830-1, 16CS1292-4 and 16CS1285-4 we detected 2, 8, 13, 14, and 63 IS elements, respectively, as well as 6, 4, 8, 6, 15 GIs, respectively. One prophage was found in three strains (16CS0369-1-AR-4, 16CS0830-1 and 16CS1285-4). Only two strains (16CS0830-1, 16CS0369-1-AR-4) carried CRISPR elements (Supplementary Table S4). Additionally, an average of 300 repeats of size >1,000 bp were detected in each chromosome. These results were also similar in the remaining fragmented genomes of cluster I (*n* = 39). However, mobile elements usually exist in multiple copies in the genome and therefore an underestimation of their occurrence in the fragmented genomes may occur. This is because Illumina sequencing is not compatible with repeats (Torresen et al., 2019). In general, the fragmented genomes (*n* = 39) carried an average of 9 (range 1–30) insertion sequences and five (range 1–10) genomic islands. Prophages and CRISPR elements were detected only in 24 and 13 genomes, respectively.

### Genetic Prediction of Antibiotic Resistance and Concordance With the Resistance Phenotype

The 27 *A. cryaerophilus* strains from Germany were susceptible to erythromycin, gentamicin and ampicillin, but were resistant to cefotaxime (Table 3). Strains 16CS1292-3 and 16CS1292-4 were resistant to tetracycline, while the strains 16CS0336-1-AR-1, 16CS0366-1-AR-2, 16CS0366-1-AR-4, and 16CS0814-1 were resistant to doxycycline. These results are in line with studies already conducted, although in these studies resistance to ampicillin has been described controversially (Ünver et al., 2013; Ferreira et al., 2016, 2019; Van den Abeele et al., 2016; Pérez-Cataluña et al., 2017). Additionally, 23 isolates showed resistance to streptomycin, while 16 strains were resistant to ciprofloxacin. This result is also largely consistent with previous studies (Van den Abeele et al., 2016; Pérez-Cataluña et al., 2017; Ferreira et al., 2019). However, previous studies used the disk diffusion method to determine antimicrobial susceptibility. Therefore, our results can be compared with those of earlier studies to a limited extent. Van den Abeele et al. (2016) compared both, the disk diffusion test and the gradient strip diffusion method. They concluded that the gradient strip method should be preferred over the disk diffusion test and that the agreement of both methods stands at 60%. This strongly argues for the need of a standardized method for measuring the antimicrobial susceptibility of *Aliarcobacter* spp. and for the evaluation of the results.

**TABLE 3:** Antimicrobial susceptibility of 27 *A. cryaerophilus* isolates.

**Isolates**	**ERY**		**CIP**		**DC**		**TC**		**GEN**		**STX**		**AMP**		**CTX**	
	**mg/L**		**mg/L**		**mg/L**		**mg/L**		**mg/L**		**mg/L**		**mg/L**		**mg/L**	
16CS0366-1-AR-1	4	S	32	R	3	R	2	S	2	S	8	R	6	S	>32	R
16CS0366-1-AR-2	4	S	32	R	3	R	2	S	2	S	8	R	5	S	>32	R
16CS0366-1-AR-3	2	S	32	R	2	S	2	S	2	S	8	R	5	S	>32	R
16CS0366-1-AR-4	4	S	32	R	3	R	2	S	2	S	8	R	5	S	>32	R
16CS0369-1-AR-1	4	S	32	R	2	S	2	S	2	S	8	R	6	S	>32	R
16CS0369-1-AR-4	2	S	32	R	2	S	2	S	2	S	8	R	4	S	>32	R
16CS0814-1	3	S	0,12	S	4	R	1	S	1,5	S	4	S	3	S	>32	R
16CS0830-1	1	S	32	R	1	S	0,38	S	0,75	S	4	S	3	S	>32	R
16CS0847-1	4	S	32	R	2	S	1,25	S	1,75	S	10	R	3	S	>32	R
16CS0847-2	2	S	16	R	1,5	S	1	S	1	S	6	R	3	S	>32	R
16CS0847-4	4	S	32	R	1,5	S	1	S	1	S	12	R	6	S	>32	R
16CS0847-5	1	S	0,12	S	0,38	S	0,5	S	1	S	6	R	3	S	>32	R
16CS0847-6	4	S	32	R	2	S	1,25	S	1,75	S	12	R	6	S	>32	R
16CS1043-1	3	S	0,06	S	2	S	1	S	1,5	S	8	R	2	S	>32	R
16CS1285-3	4	S	0,12	S	1	S	1	S	1	S	4	S	2	S	>32	R
16CS1285-4	2	S	32	R	2	S	2	S	1	S	12	R	4	S	>32	R
16CS1290-1	4	S	32	R	1,5	S	1,5	S	1,5	S	12	R	4	S	>32	R
16CS1292-3	4	S	32	R	1,5	S	16	R	1,5	S	12	R	6	S	>32	R
16CS1292-4	4	S	32	R	2	S	12	R	2	S	12	R	1,5	S	>32	R
17CS0830-1	4	S	0,12	S	1,5	S	1	S	1	S	4	S	3	S	>32	R
17CS0996-A	4	S	0,06	S	1	S	1	S	1	S	6	R	6	S	>32	R
17CS0996-B	2	S	0,06	S	1,5	S	1	S	1	S	6	R	4	S	>32	R
17CS1055-A	2	S	0,12	S	2	S	0,25	S	0,5	S	8	R	6	S	>32	R
17CS1055-B	1	S	8	R	1	S	1	S	1	S	12	R	2	S	>32	R
17CS1061	1	S	0,06	S	0,75	S	0,5	S	1	S	6	R	2	S	>32	R
17CS1201-1	2	S	0,12	S	2	S	1	S	1	S	6	R	1,5	S	8	R
17CS1201-2	0,5	S	0,06	S	0,75	S	0,5	S	2	S	6	R	3	S	>32	R

*CIP – ciprofloxacin, DC – doxycycline, TC – tetracycline, GEN – gentamicin, STX – streptomycin, AMP – ampicillin, CTX – cefotaxime, S – susceptible, R – resistant.*

**FIGURE 4:**
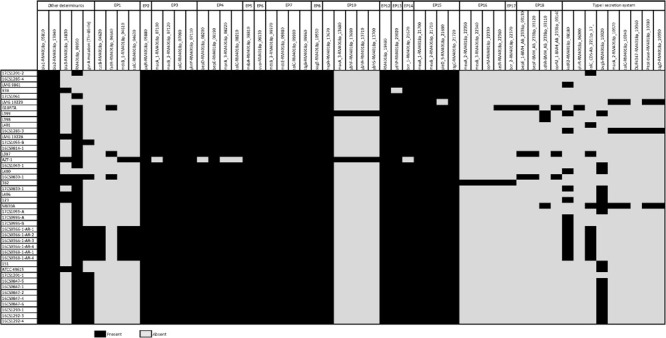
Predicted antimicrobial resistance determinants in 44 *A. cryaerophilus* genomes of cluster I including 16 efflux pumps (EP) systems, a type I secretion system (T1SS), resistance genes and the *gyr*A mutation (Thr-85-Ile).

Utilizing the genomes of cluster I the genetic prediction of antimicrobial resistance genes was done using the custom database created by Isidro et al. (2020). Out of 19 efflux pump (EP) systems which have been detected in *A. butzleri* genomes, 16 were found in the *A. cryaerophilus* genomes belonging to cluster I (*n* = 44) (Figure 4). The three missing efflux pump systems are EP9, EP11, and EP19. Six EP systems were present in all genomes: (a) EP2 and EP12 [both belong to the major facilitator superfamily (MFS)]; (b) EP5 and EP6 [both belong to the ATP-binding cassette (ABC) superfamily]; (c) EP7 [belongs to the resistance-nodulation-division (RND) family]; and (d) EP8 [belongs to the small multidrug resistance (SMR) family]. The remaining ten EP systems belong to the RND, ABC and MFS families and were present at least in one strain. Those findings showed that *A. cryaerophilus* harbors all major families of efflux transporters that are present in prokaryotes apart from the multidrug and toxic efflux (MATE) family (Webber and Piddock, 2003). Since the protein size of regulator TetR (RM4018p_22360) from EP16 is supposed to correlate with the erythromycin resistance, Isidro et al. (2020) hypothesized that truncating mutations in TetR lead to an overexpression of EP16 and thus to increased erythromycin excretion and ultimately cause resistance or tolerance to this antibiotic. The regulator TetR was not present in our 27 German *A. cryaerophilus* strains, which were all susceptible to erythromycin. EP3, a member of the ABC family, might also be involved in erythromycin resistance as it contains *mac*A and *mac*B genes that encode macrolide export proteins (Fanelli et al., 2019). Although both genes were found in all German strains, the strains were susceptible to erythromycin.

A complete type I secretion system (T1SS) was not detected in any of the 44 *A. cryaerophilus* genomes of cluster I. Apart from the fact that only six instead of seven genes were detected (DNA-binding response regulator gene (RM4018p_10330) was missing), these genes were not present in all strains. 16CS1285-3 was the only genome that carried five T1SS genes.

The search for other antimicrobial resistance determinants revealed that all 44 *A. cryaerophilus* strains harbor two beta-lactamase genes (*bla*1, RM4018p_05810; *bla*2, RM4018p_13040), while eight isolates also carried *bla*3 (RM4018p_14830). None of the strains carried the chloramphenicol acetyltransferase gene (*cat*3, RM4018p_07870). Isidro et al. (2020) detected a strong correlation between the presence of an OXA-15-like beta-lactamase gene (*bla*3) and ampicillin resistance. This beta-lactamase gene was detected in one strain (16CS0830-1). Contradictory, the strain was phenotypically susceptible to ampicillin *in vitro*. This may indicate that not only the presence of the beta-lactamase gene is important, but also its activity together with penicillin-binding proteins and outer-membrane permeability (Fanelli et al., 2019). The presence of the two beta-lactamase genes *bla*1 and *bla*2 in all 44 genomes might be the reason for the cefotaxime resistance. Resistance to ciprofloxacin in *Aliarcobacter* spp. is, as reported previously, caused by a point mutation in the quinolone resistance determining region (QRDR) at position 254 of the *gyr*A gene (Abdelbaqi et al., 2007). This mutation subsequently leads to an amino acid exchange from threonine to isoleucine (Thr-85-Ile). In this study, out of 16 resistant *A. cryaerophilus* strains, 15 exhibited this mutation (Supplementary Table S5). One strain, 16CS1285-4, was phenotypically resistant but did not carry this specific mutation or any other known mutation [e.g., Asp-89-Tyr (Ferreira et al., 2018)]. Interestingly, two susceptible strains had the reported mutation. These observations show that not in every case resistance to ciprofloxacin is due to a single mutation in the *gyr*A gene in *A. cryaerophilus*. Maybe a functional multidrug efflux pump e.g., *cme*ABC (RND) is also required as described before for *Campylobacter* (Shen et al., 2018). It is noteworthy, that the topoisomerase IV genes *par*C and *par*E which are also responsible for fluoroquinolone resistance were absent in the tested strains, suggesting that they are not involved in ciprofloxacin resistance in *A. cryaerophilus*.

Three of our four plasmid sequences carried at least one gene that is associated with antimicrobial resistance e.g., multidrug efflux systems *cme*ABC and *cme*DEF (RND) and a streptomycin-3-O-adenyltransferase (Supplementary Table S6), showing that *A. cryaerophilus* may be able to exchange antimicrobial resistance genes.

### *In silico* Virulence Gene Profiling

As with antibiotic resistance genes, the database created by Isidro et al. (2020) was also used to identify potential virulence determinants in *A. cryaerophilus* genomes of cluster I. This database includes genes for the flagellum synthesis, chemotaxis system, and capsule as well as genes for adherence, invasion, iron uptake, type IV secretion system (T4SS), and an urease cluster.

**FIGURE 5:**
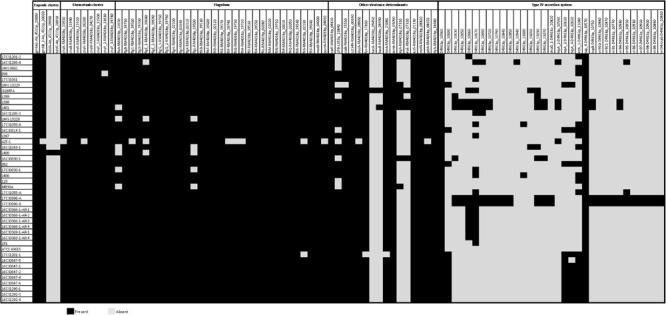
Predicted virulence-associated determinants in 44 *A. cryaerophilus* genomes including 4 capsule genes, 8 chemotaxis system genes, 31 flagellar genes, 33 type IV secretion system genes, and other virulence determinants genes.

The survey showed that none of the 44 genomes carried the urease cluster (Figure 5). This finding was not surprising because *A. cryaerophilus* is reported to be non-ureolytic compared to *A. butzleri* (Miller et al., 2018).

Furthermore, only fragments of the T4SS were found, since 33 of 55 genes were detected in our strains. These 33 genes were either present in all isolates (e.g., PAS domain-containing protein, D4963p_10560) or just in one strain (e.g., hypothetical protein, D4963p_10940).

The potential capsule cluster was only found partially. Four out of 7 genes, namely *gmh*A2, *gmh*B, *hdd*A, and *hdd*C, were detected. While strain L400 (accession: GCF_001573005.1) carried all 4 genes, the *gmh*A2 gene was the only gene present in all tested genomes.

*Aliarcobacter cryaerophilus* is a motile bacterium with a polar flagellum like *A. butzleri*. It was therefore not surprising that flagellum genes were detectable. However, three genes, namely *fla*B (minor flagellin subunit), *flh*A (flagellar biosynthesis protein), and *hag* (another gene encoding flagellin), out of 34 flagellar genes could not be found in any of the 44 genomes. Of the remaining 31 genes, 22 were detected in all isolates, with strain AZT-1 having the fewest flagellum genes. Nevertheless, the missing flagellar genes could have an impact on the assembly or function of the flagellum.

We highlight the presence of the chemotaxis system genes (*che*A-*che*Y) in all tested *A. cryaerophilus* isolates apart from the strains AZT-1 and 938. While *che*Y2 was not found in strain 938, the genes *che*A and *che*V were not found in strain AZT-1. This result shows that *A. butzleri* is not the only *Aliarcobacter* spp. that carries a full chemotaxis system (Miller et al., 2007; Isidro et al., 2020). Interestingly, the chemotaxis-associated genes *doc*A and *lux*S were also present in all investigated isolates. Only strain AZT-1 had no *doc*A gene.

The following virulence determinants associated with cellular adhesion and invasion were present in all 44 genomes: *cad*F, *cj*1349 (both fibronectin-binding proteins), *tly*A (hemolysin), *iam*A (invasion-associated gene), *mvi*N (inner membrane protein for peptidoglycan biosynthesis). The genes *cia*B (host cell invasion) and *pld*A (outer membrane phospholipase A) were present in all strains except in LMG 10229^T^ and AZT-1, respectively. The gene *htr*A–a chaperon involved in adhesins folding (Isidro et al., 2020) – was present in 43 isolates, but not in strain 17CS1201-1. While *hec*A (filamentous hemagglutinin) was only present in G18RTA (accession: GCF_008086605.1), *hec*B (hemolysin activation protein) occurred in both, G18RTA and L401. Of the two genes, namely *cfr*B and *fur*, possibly involved in iron uptake, *fur* was detected in all strains but not in 17CS1201-1. The *cfr*B gene was found to be present in 24 isolates. The genes *irg*A and *iro*E which have been associated with the uropathogenicity of *E. coli* (Miller et al., 2007) were both detected in five genomes, with *irg*A being present in three additional strains.

Previous studies reported that the virulence genes *cia*B, *cad*F, *cj*1349, *pld*A, and *mvi*N are more frequently detected in *A. cryaerophilus* strains than the other virulence determinants e.g., *tly*A, *irg*A, iroE, *hec*A, and *hec*B (Douidah et al., 2012; Zacharow et al., 2015; Sekhar et al., 2017; Brückner et al., 2020). This is mostly in line with our data. Here, only strain G18RTA carried all 14 virulence genes associated with adherence, invasion, and iron uptake. This is consistent with previous reports, as it is very rare to find all virulence genes in every *A. cryaerophilus* isolate (Zacharow et al., 2015; Sekhar et al., 2017; Brückner et al., 2020). In fact, previous studies reported that the phenotypic urease test in *A. cryaerophilus* was negative. Therefore it could be hypothesized that *A. cryaerophilus* is not able to metabolize urea and may not be able to survive in acidic surroundings, e.g., in the urinary tract (Pérez-Cataluña et al., 2018b, 2019).

Additionally, the findings of the virulence-associated genes – previously found on the virulence plasmid from an *A. cryaerophilus* strain (BankIt2207814 M830MA_plasmid MK715471) (On et al., 2019) – in all *A. cryaerophilus* sequences used in the present study are summarized in Supplementary Table S7. While one virulence-associated gene was found in 14 strains, five strains carried two genes. These findings indicate that *A. cryaerophilus* may also be able to exchange virulence factors.

Although *A. cryaerophilus* has a large repertoire of virulence genes, the investigated strains were isolated from healthy animals, probably indicating that a complex mechanism of virulence exists and that the sole presence of *A. cryaerophilus* in the gut may not be sufficient for pathogenicity.

## Conclusion

To the best of our knowledge, this study presents the first report of whole-genome sequencing data of *A. cryaerophilus* from Germany. The genomic information on *A. cryaerophilus* is currently limited and as of to date no in-depth comparative genomic analysis has been conducted. In this study, a total of 27 *A. cryaerophilus* strains were isolated from seven poultry farms in Germany. These strains belong to the *A. cryaerophilus* cluster I following a recent taxonomic proposal. A high genetic diversity between *A. cryaerophilus* from Germany was observed. This is despite that the strains were restricted to a single federal state (Thuringia, Germany), a particular host (water poultry), and were collected over a short period of time (2 years). Additionally, the phylogenetic analysis of cluster I did not support distinct clustering based on the geographical or ecological niche of the strains.

The genomic features of cluster I *A. cryaerophilus* strains include: a chromosome densely packed with coding sequences (91.5–93.4% of the chromosome size); variable presence of mobile elements such as IS, GI and prophages. Furthermore, the alignment of the chromosomes from our four circularized genomes compared with the genome of strain ATCC 49615 revealed a considerable degree of synteny, however, inversion and shifts were observed.

The genetic prediction of virulence and antibiotic resistance showed that *A. cryaerophilus* has a large virulome and that the genetic antimicrobial resistance is only to a limited extent consistent with the phenotypic characterization. Therefore, antimicrobial susceptibility should continue to be tested phenotypically. Surprisingly, *A. cryaerophilus* appears to be more resistant to ciprofloxacin than *A. butzleri*. Although a partial correlation has been found between the presence of the *gyr*A mutation (Thr-85-Ile) and ciprofloxacin resistance, this does not apply to all resistant strains. It is noteworthy, that a functional multidrug efflux pump like *cme*ABC could also be a possible resistance mechanism against ciprofloxacin. The presence of two beta-lactamases (*bla*1, *bla*2) in all *A. cryaerophilus* genomes from cluster I may correlate with the resistance to cefotaxime. Additionally, to our knowledge, this is the first report of the detection of antimicrobial resistance determinants in *A. cryaerophilus* plasmids, which indicates the possibility of exchanging resistance genes between different strains.

## Data Availability Statement

The original contributions presented in this study are publicly available. This data can be found here: DDBJ/ENA/GenBank; BioProject: PRJNA632720. Publicly available datasets were analyzed in this study. This data can be found here: DDBJ/ENA/GenBank; BioProject: PRJNA66819, PRJNA302819, PRJNA307600, PRJNA294644, PRJNA294645, PRJNA294646, PRJNA308312, PRJNA294642, PRJNA369468, and PRJNA431460.

## Author Contributions

EM, HT, and MA-G designed the work. CA provided the fecal samples and metadata. EM, IH, and MA-G performed the analyses. EM and MA-G interpreted the data and wrote the manuscript. HH and HT supervised the analyses. All authors contributed to the revision of the manuscript, read, and approved the submitted manuscript.

## Conflict of Interest

The authors declare that the research was conducted in the absence of any commercial or financial relationships that could be construed as a potential conflict of interest.

## Footnotes

1 https://github.com/tseemann/shovill

2 https://github.com/nanoporetech/medaka

3 https://github.com/tseemann/barrnap

4 https://github.com/tseemann/mlst

5 https://github.com/tseemann/abricate

6 https://www.ncbi.nlm.nih.gov/genome/browse/#!/prokaryotes/11530/

## References

[B1] Abdelbaqi K. Menard A. Prouzet-Mauleon V. Bringaud F. Lehours P. Megraud F. (2007). Nucleotide sequence of the *gyrA* gene of *Arcobacter* species and characterization of human ciprofloxacin-resistant clinical isolates. *FEMS Immunol. Med. Microbiol.* 49 337–345. 10.1111/j.1574-695X.2006.00208.x 17378897

[B2] Altschul S. F. Gish W. Miller W. Myers E. W. Lipman D. J. (1990). Basic local alignment search tool. *J. Mol. Biol.* 215 403–410. 10.1016/S0022-2836(05)80360-2 2231712

[B3] Atabay H. I. Unver A. Sahin M. Otlu S. Elmali M. Yaman H. (2008). Isolation of various *Arcobacter* species from domestic geese (*Anser anser*). *Vet. Microbiol.* 128 400–405. 10.1016/j.vetmic.2007.10.010 18023541

[B4] Benson D. A. Cavanaugh M. Clark K. Karsch-Mizrachi I. Lipman D. J. Ostell J. (2013). GenBank. *Nucleic Acids Res.* 41 D36–D42. 10.1093/nar/gks1195 23193287 PMC3531190

[B5] Bertelli C. Laird M. R. Williams K. P. Simon Fraser University Research Computing Group Lau B. Y. Hoad G. (2017). IslandViewer 4: expanded prediction of genomic islands for larger-scale datasets. *Nucleic Acids Res.* 45 W30–W35. 10.1093/nar/gkx343 28472413 PMC5570257

[B6] Brückner V. Fiebiger U. Ignatius R. Friesen J. Eisenblatter M. Hock M. (2020). Characterization of *Arcobacter* strains isolated from human stool samples: results from the prospective German prevalence study Arcopath. *Gut Pathog.* 12:3. 10.1186/s13099-019-0344-3 31921357 PMC6947975

[B7] Collado L. Figueras M. J. (2011). Taxonomy, epidemiology, and clinical relevance of the genus *Arcobacter*. *Clin. Microbiol. Rev.* 24 174–192. 10.1128/CMR.00034-10 21233511 PMC3021208

[B8] Darling A. E. Mau B. Perna N. T. (2010). progressiveMauve: multiple genome alignment with gene gain, loss and rearrangement. *PLoS One* 5:e11147. 10.1371/journal.pone.0011147 20593022 PMC2892488

[B9] Debruyne L. Houf K. Douidah L. De Smet S. Vandamme P. (2010). Reassessment of the taxonomy of *Arcobacter cryaerophilus*. *Syst. Appl. Microbiol.* 33 7–14. 10.1016/j.syapm.2009.10.001 19945242

[B10] Douidah L. de Zutter L. Bare J. De Vos P. Vandamme P. Vandenberg O. (2012). Occurrence of putative virulence genes in *Arcobacter* species isolated from humans and animals. *J. Clin. Microbiol.* 50 735–741. 10.1128/JCM.05872-11 22170914 PMC3295157

[B11] Douidah L. De Zutter L. Van Nieuwerburgh F. Deforce D. Ingmer H. Vandenberg O. (2014). Presence and analysis of plasmids in human and animal associated *Arcobacter* species. *PLoS One* 9:e85487. 10.1371/journal.pone.0085487 24465575 PMC3896396

[B12] El-Ashker M. Gwida M. Tomaso H. Monecke S. Ehricht R. El-Gohary F. (2015). Staphylococci in cattle and buffaloes with mastitis in Dakahlia Governorate. *Egypt. J. Dairy Sci.* 98 7450–7459. 10.3168/jds.2015-9432 26364099

[B13] EUCAST, (2019). *Breakpoint Tables for Interpretation of MICs and Zone Diameters. Version 9.0, 2019.* Sweden: The European Committee on Antimicrobial Susceptibility Testing.

[B14] European Food Safety Authority Aerts M. Battisti A. Hendriksen R. Kempf I. Teale C. (2019). Technical specifications on harmonised monitoring of antimicrobial resistance in zoonotic and indicator bacteria from food-producing animals and food. *EFSA J.* 17:e05709. 10.2903/j.efsa.2019.5709 32626332 PMC7009308

[B15] Fanelli F. Di Pinto A. Mottola A. Mule G. Chieffi D. Baruzzi F. (2019). Genomic characterization of *Arcobacter butzleri* isolated from shellfish: novel insight into antibiotic resistance and virulence determinants. *Front. Microbiol.* 10:670. 10.3389/fmicb.2019.00670 31057492 PMC6477937

[B16] Feldgarden M. Brover V. Haft D. H. Prasad A. B. Slotta D. J. Tolstoy I. (2019). Using the NCBI AMRFinder tool to determine antimicrobial resistance genotype-phenotype correlations within a collection of NARMS isolates. *bioRxiv.* [Preprint]. 10.1101/550707 %J bioRxiv

[B17] Ferreira S. Correia D. R. Oleastro M. Domingues F. C. (2018). *Arcobacter* butzleri ciprofloxacin resistance: point mutations in dna gyrase a and role on fitness cost. *Microb. Drug Resist.* 24 915–922. 10.1089/mdr.2017.0295 29336679

[B18] Ferreira S. Luis A. Oleastro M. Pereira L. Domingues F. C. (2019). A meta-analytic perspective on *Arcobacter* spp. antibiotic resistance. *J. Glob. Antimicrob. Resist.* 16 130–139. 10.1016/j.jgar.2018.12.018 30611931

[B19] Ferreira S. Queiroz J. A. Oleastro M. Domingues F. C. (2016). Insights in the pathogenesis and resistance of *Arcobacter*: A review. *Crit. Rev. Microbiol.* 42 364–383. 10.3109/1040841X.2014.954523 25806423

[B20] Gaiarsa S. Batisti Biffignandi G. Esposito E. P. Castelli M. Jolley K. A. Brisse S. (2019). Comparative analysis of the two *Acinetobacter baumannii* multilocus sequence typing (MLST) schemes. *Front. Microbiol.* 10:930. 10.3389/fmicb.2019.00930 31130931 PMC6510311

[B21] Gupta S. K. Padmanabhan B. R. Diene S. M. Lopez-Rojas R. Kempf M. Landraud L. (2014). ARG-ANNOT, a new bioinformatic tool to discover antibiotic resistance genes in bacterial genomes. *Antimicrob. Agents Chemother.* 58 212–220. 10.1128/AAC.01310-13 24145532 PMC3910750

[B22] Hänel I. Hotzel H. Tomaso H. Busch A. (2018). Antimicrobial susceptibility and genomic Structure of *Arcobacter skirrowii* Isolates. *Front. Microbiol.* 9:3067. 10.3389/fmicb.2018.03067 30619152 PMC6302008

[B23] Harrass B. Schwarz S. Wenzel S. (1998). Identification and characterization of *Arcobacter* isolates from broilers by biochemical tests, antimicrobial resistance patterns and plasmid analysis. *Zentralbl Veterinarmed B* 45 87–94. 10.1111/j.1439-0450.1998.tb00770.x 9557130

[B24] Ho H. T. Lipman L. J. Gaastra W. (2008). The introduction of *Arcobacter* spp. in poultry slaughterhouses. *Int. J. Food Microbiol.* 125 223–229. 10.1016/j.ijfoodmicro.2008.02.012 18579247

[B25] Ho H. T. K. Lipman L. J. A. Gaastra W. (2006). *Arcobacter*, what is known and unknown about a potential foodborne zoonotic agent! *Vet. Microbiol.* 115 1–13. 10.1016/j.vetmic.2006.03.004 16621345

[B26] Houf K. Tutenel A. De Zutter L. Van Hoof J. Vandamme P. (2000). Development of a multiplex PCR assay for the simultaneous detection and identification of *Arcobacter butzleri*, *Arcobacter cryaerophilus* and *Arcobacter skirrowii*. *FEMS Microbiol. Lett.* 193 89–94. 10.1111/j.1574-6968.2000.tb09407.x 11094284

[B27] Hsueh P. R. Teng L. J. Yang P. C. Wang S. K. Chang S. C. Ho S. W. (1997). Bacteremia caused by *Arcobacter cryaerophilus* 1B. *J. Clin. Microbiol.* 35 489–491. 10.1128/jcm.35.2.489-491.1997 9003624 PMC229608

[B28] ICMSF, (2002). *Microorganisms in Foods 7. Microbiological Testing in Food Safety Management.* New York, NY: Kluwer Academic/Plenum Publishers.

[B29] Isidro J. Ferreira S. Pinto M. Domingues F. Oleastro M. Gomes J. P. (2020). Virulence and antibiotic resistance plasticity of *Arcobacter butzleri*: Insights on the genomic diversity of an emerging human pathogen. *Infect. Genet. Evol.* 80:104213. 10.1016/j.meegid.2020.104213 32006709

[B30] Jia B. Raphenya A. R. Alcock B. Waglechner N. Guo P. Tsang K. K. (2017). CARD 2017: expansion and model-centric curation of the comprehensive antibiotic resistance database. *Nucleic Acids Res.* 45 D566–D573. 10.1093/nar/gkw1004 27789705 PMC5210516

[B31] Jolley K. A. Maiden M. C. J. (2010). BIGSdb: Scalable analysis of bacterial genome variation at the population level. *BMC Bioinformatics* 11:595. 10.1186/1471-2105-11-595 21143983 PMC3004885

[B32] Katoh K. Misawa K. Kuma K. Miyata T. (2002). MAFFT: a novel method for rapid multiple sequence alignment based on fast Fourier transform. *Nucleic Acids Res.* 30 3059–3066. 10.1093/nar/gkf436 12136088 PMC135756

[B33] Kearse M. Moir R. Wilson A. Stones-Havas S. Cheung M. Sturrock S. (2012). Geneious Basic: an integrated and extendable desktop software platform for the organization and analysis of sequence data. *Bioinformatics* 28 1647–1649. 10.1093/bioinformatics/bts199 22543367 PMC3371832

[B34] Kiehlbauch J. A. Plikaytis B. D. Swaminathan B. Cameron D. N. Wachsmuth I. K. (1991). Restriction fragment length polymorphisms in the ribosomal genes for species identification and subtyping of aerotolerant *Campylobacter* species. *J. Clin. Microbiol.* 29 1670–1676. 10.1128/jcm.29.8.1670-1676.1991 1684797 PMC270182

[B35] Kolmogorov M. Yuan J. Lin Y. Pevzner P. A. (2019). Assembly of long, error-prone reads using repeat graphs. *Nat. Biotechnol.* 37 540–546. 10.1038/s41587-019-0072-8 30936562

[B36] Kumar S. Stecher G. Li M. Knyaz C. Tamura K. (2018). MEGA X: molecular evolutionary genetics analysis across computing platforms. *Mol. Biol. Evol.* 35 1547–1549. 10.1093/molbev/msy096 29722887 PMC5967553

[B37] Meier-Kolthoff J. P. Auch A. F. Klenk H. P. Goker M. (2013). Genome sequence-based species delimitation with confidence intervals and improved distance functions. *BMC Bioinformatics* 14:60. 10.1186/1471-2105-14-60 23432962 PMC3665452

[B38] Millar J. A. Raghavan R. (2017). Accumulation and expression of multiple antibiotic resistance genes in *Arcobacter cryaerophilus* that thrives in sewage. *PeerJ* 5:e3269. 10.7717/peerj.3269 28462059 PMC5407278

[B39] Miller W. G. Parker C. T. Rubenfield M. Mendz G. L. Wosten M. M. Ussery D. W. (2007). The complete genome sequence and analysis of the epsilonproteobacterium *Arcobacter butzleri*. *PLoS One* 2:e1358. 10.1371/journal.pone.0001358 18159241 PMC2147049

[B40] Miller W. G. Yee E. Bono J. L. (2018). Complete Genome Sequences of the *Arcobacter cryaerophilus* Strains ATCC 43158(T) and ATCC 49615. *Microbiol Resour Announc* 7:e01463-18. 10.1128/MRA.01463-18 30533823 PMC6256622

[B41] On S. L. W. Althaus D. Miller W. G. Lizamore D. Wong S. G. L. Mathai A. J. (2019). *Arcobacter* cryaerophilus isolated from New Zealand mussels harbor a putative virulence plasmid. *Front. Microbiol.* 10:1802. 10.3389/fmicb.2019.01802 31428079 PMC6690266

[B42] Page A. J. Cummins C. A. Hunt M. Wong V. K. Reuter S. Holden M. T. (2015). Roary: rapid large-scale prokaryote pan genome analysis. *Bioinformatics* 31 3691–3693. 10.1093/bioinformatics/btv421 26198102 PMC4817141

[B43] Parkhill J. Wren B. W. Mungall K. Ketley J. M. Churcher C. Basham D. (2000). The genome sequence of the food-borne pathogen *Campylobacter jejuni* reveals hypervariable sequences. *Nature* 403 665–668. 10.1038/35001088 10688204

[B44] Pearson W. R. (2013). An introduction to sequence similarity (“homology”) searching. *Curr. Protoc. Bioinformatics* Chapter 3 Unit31. 10.1002/0471250953.bi0301s42 23749753 PMC3820096

[B45] Pérez-Cataluña A. Collado L. Salgado O. Lefiñanco V. Figueras M. J. (2018a). A polyphasic and taxogenomic evaluation uncovers *Arcobacter cryaerophilus* as a species complex that embraces four genomovars. 9:805. 10.3389/fmicb.2018.00805 29755434 PMC5934430

[B46] Pérez-Cataluña A. Salas-Masso N. Dieguez A. L. Balboa S. Lema A. Romalde J. L. (2018b). Revisiting the taxonomy of the genus *Arcobacter*: getting order from the chaos. *Front. Microbiol.* 9:2077. 10.3389/fmicb.2018.02077 30233547 PMC6131481

[B47] Pérez-Cataluña A. Salas-Masso N. Dieguez A. L. Balboa S. Lema A. Romalde J. L. (2019). Corrigendum (2): revisiting the taxonomy of the genus *Arcobacter*: getting order from the chaos. *Front. Microbiol.* 10:2253. 10.3389/fmicb.2019.02253 31611866 PMC6779803

[B48] Pérez-Cataluña A. Tapiol J. Benavent C. Sarvise C. Gomez F. Martinez B. (2017). Antimicrobial susceptibility, virulence potential and sequence types associated with *Arcobacter* strains recovered from human faeces. *J. Med. Microbiol.* 66 1736–1743. 10.1099/jmm.0.000638 29120301

[B49] Pritchard L. Glover R. H. Humphris S. Elphinstone J. G. Toth I. K. (2016). Genomics and taxonomy in diagnostics for food security: soft-rotting enterobacterial plant pathogens. *Anal. Methods* 8 12–24. 10.1039/C5AY02550H

[B50] Ramees T. P. Dhama K. Karthik K. Rathore R. S. Kumar A. Saminathan M. (2017). *Arcobacter*: an emerging food-borne zoonotic pathogen, its public health concerns and advances in diagnosis and control–a comprehensive review. *Vet. Q.* 37 136–161. 10.1080/01652176.2017.1323355 28438095

[B51] Seemann T. (2014). Prokka: rapid prokaryotic genome annotation. *Bioinformatics* 30 2068–2069. 10.1093/bioinformatics/btu153 24642063

[B52] Sekhar M. S. Tumati S. R. Chinnam B. K. Kothapalli V. S. Sharif N. M. (2017). Virulence gene profiles of *Arcobacter* species isolated from animals, foods of animal origin, and humans in Andhra Pradesh, India. *Vet. World* 10 716–720. 10.14202/vetworld.2017.716-720 28717327 PMC5499092

[B53] Shen Z. Wang Y. Zhang Q. Shen J. (2018). Antimicrobial resistance in *Campylobacter* spp. *Microbiol. Spectr.* 6 317–330. 10.1128/microbiolspec.ARBA-0013-2017 29623873 PMC11633568

[B54] Song W. Sun H. X. Zhang C. Cheng L. Peng Y. Deng Z. (2019). Prophage hunter: an integrative hunting tool for active prophages. *Nucleic Acids Res.* 47 W74–W80. 10.1093/nar/gkz380 31114893 PMC6602508

[B55] Torresen O. K. Star B. Mier P. Andrade-Navarro M. A. Bateman A. Jarnot P. (2019). Tandem repeats lead to sequence assembly errors and impose multi-level challenges for genome and protein databases. *Nucleic Acids Res.* 47 10994–11006. 10.1093/nar/gkz841 31584084 PMC6868369

[B56] Treangen T. J. Ondov B. D. Koren S. Phillippy A. M. (2014). The Harvest suite for rapid core-genome alignment and visualization of thousands of intraspecific microbial genomes. *Genome Biol.* 15:524. 10.1186/s13059-014-0524-x 25410596 PMC4262987

[B57] Ünver A. H. Ý Çahin M. ÇelebiÝ Ö (2013). Antimicrobial susceptibilities of various *Arcobacter* species. *Turk. J. Med. Sci.* 43 548–552. 10.3906/sag-1207-115 31411186

[B58] Van den Abeele A. M. Vogelaers D. Vanlaere E. Houf K. (2016). Antimicrobial susceptibility testing of *Arcobacter butzleri* and *Arcobacter cryaerophilus* strains isolated from Belgian patients. *J. Antimicrob. Chemother.* 71 1241–1244. 10.1093/jac/dkv483 26851610

[B59] Vandamme P. Vancanneyt M. Pot B. Mels L. Hoste B. Dewettinck D. (1992). Polyphasic taxonomic study of the emended genus *Arcobacter* with *Arcobacter butzleri* comb. nov. and *Arcobacter skirrowii* sp. nov., an aerotolerant bacterium isolated from veterinary specimens. *Int. J. Syst. Bacteriol.* 42 344–356. 10.1099/00207713-42-3-344 1503968

[B60] Vandenberg O. Dediste A. Houf K. Ibekwem S. Souayah H. Cadranel S. (2004). *Arcobacter* species in humans. *Emerg Infect. Dis.* 10 1863–1867. 10.3201/eid1010.040241 15504280 PMC3323243

[B61] Vaser R. Sovic I. Nagarajan N. Sikic M. (2017). Fast and accurate *de novo* genome assembly from long uncorrected reads. *Genome Res.* 27 737–746. 10.1101/gr.214270.116 28100585 PMC5411768

[B62] Walker B. J. Abeel T. Shea T. Priest M. Abouelliel A. Sakthikumar S. (2014). Pilon: an integrated tool for comprehensive microbial variant detection and genome assembly improvement. *PLoS One* 9:e112963. 10.1371/journal.pone.0112963 25409509 PMC4237348

[B63] Webber M. A. Piddock L. J. (2003). The importance of efflux pumps in bacterial antibiotic resistance. *J. Antimicrob. Chemother.* 51 9–11. 10.1093/jac/dkg050 12493781

[B64] Xie Z. Tang H. (2017). ISEScan: automated identification of insertion sequence elements in prokaryotic genomes. *Bioinformatics* 33 3340–3347. 10.1093/bioinformatics/btx433 29077810

[B65] Zacharow I. Bystron J. Walecka-Zacharska E. Podkowik M. Bania J. (2015). Genetic diversity and incidence of virulence-associated genes of *Arcobacter butzleri* and *Arcobacter cryaerophilus* isolates from pork, beef, and chicken meat in poland. *Biomed. Res. Int.* 2015:956507. 10.1155/2015/956507 26539546 PMC4619883

[B66] Zankari E. Hasman H. Cosentino S. Vestergaard M. Rasmussen S. Lund O. (2012). Identification of acquired antimicrobial resistance genes. *J. Antimicrob. Chemother.* 67 2640–2644. 10.1093/jac/dks261 22782487 PMC3468078

